# Notes and News

**Published:** 1893-05-06

**Authors:** 


### NOTES AND NEWS.

COLLECTED AND COMMUNICATED.

Saturday, June 24th, is the date now officially fixed for the opening of the new wing at the Hospital for Sick Children, Great Ormond Street, by the Prince and Princess of Wales. The Princess of Wales will receive purses towards the building fund, which amounts to £12,000.

A French medical contemporary has much to say on the subject of bicycling, and quotes most contradictory opinions for and against its value as a health-giving exercise. On the whole, French opinion is in favour of cycling, and we are told in rough numbers that there are now 300,000 cyclists in France. Viewed in the light of a distinctly beneficial exercise, it is a little hard that a tax should have been placed on bicycles from over the water, thus preventing many a young Englishman, to whom every shilling spent on the holiday is of consequence, from exploring the many lovely spots in France only possible with the aid of the bicycle.

There is much force in an effective appeal. We would recommend to the notice of hospital secretaries one we received from the Rector of Kelvedon Hatch, Brentwood, which runs as follows:—

Dear Sir,—On opening this letter your thoughts will perhaps turn to the waste paper basket, and in nine cases out of ten such letters find a resting-place there, almost before they are opened. Now, I do not plead for this letter any better resting-place than its fellows; but I do plead for it this exceptional favour, that you will kindly read it through first. I would not even give you this trouble were it not for the fact that our needs are so very real and urgent, and we have nowhere to look to for help.

Mr. Peregrine's letter is followed by a statement of the need of a church for his parish. The parish is an almost entirely poor one, and the Rector is forced to appeal outside it. The Bishops of St. Albans and Colchester warmly support him, but the contribution list at present mainly consists of donations of members of Mr. Peregrine's own family. But that was before the publication of his effective letter.

Earlswood Asylum is a familiar name to us, and to most of us little more than a name of disagreeable associations. An interesting little article in *Religious Bits* shows us that Earlswood is not without its bright side. The brightness is perhaps chiefly contributed by the youthful inmates, and the improvement in the whole condition of children after admission is very marked, even if entire cure is not effected. All that can be done to brighten these afflicted young lives is carefully carried out. Lessons are rendered bright and pleasant, and mind and body further kept occupied by the useful pursuit of some trade or employment. Each child is taught to know its own bed by a doll which rests on each pillow, the dolls being rendered as distinct from one another as possible. They are a source of comfort and amusement to the children. Both for young and old the establishment is rendered as attractive as possible, and kind friends have presented pictures and ornaments to decorate the apartments. It is suggested, and we think the idea should certainly be carried out, that each parent whose child is restored in health should make a point of becoming an annual subscriber, if only of 10s. It must be remembered that the children do not return to their parents after a period spent in their cure only, but have mostly acquired technical skill of some kind, often in advance of any that could have been attained in their own homes. It is interesting to know that all the printing required by the institution is skilfully executed by the inmates.

Mr. William Freshfield writes on behalf of the Royal Hospital for Incurables, Putney: "The institution at West Hill, Putney, is now so well know, and has done, and is doing, so good a work, that it is almost superfluous to do more than ask for subscriptions. In charity, however, as in other matters, competition is so fierce, that even the most popular and best regulated and managed hospitals cannot rely solely on their character. This being so, I urgently press on you to investigate for yourself the claims of this charity. Shortly, the work which the hospital does is this: It provides pensions of £20 a-year for those who are absolutely incurable, and who are deserving of help, and whose relations cannot afford to support them. These pensions enable the recipents to maintain the ties of domestic life, though the comforts which they would receive at their own homes might not be equal to those which they would receive if they became inmates. They suffice, however, for the state which they prefer. The hospital further provides a home for incurables—persons who, having outlived their friends and their means, and not being of the pauper class, but being absolutely helpless and incurable, have the worst of fates before them. To these the Royal Hospital for Incurables stretches out a helping hand and gives a home for life. In this home no question of religious persuasion is allowed to intervene; the only object of the institution is to lighten the burden of pain which must be borne by those who are incurable and cannot help themselves, and have no friends to help them. Attached to the head institution is a seaside house at St. Leonard's, to which the inmates of the hospital itself are sent under medical advice. At present there are 588 pensioners and 209 inmates. Is it necessary for me to do more than to state these facts to ensure your cordial co-operation in this noble work. Kindly send your reply to the Secretary, at 106, Queen Victoria Street, E.C."

